# ‘Guide and Prejudice’— How Argonautes recognize targets across domains of life

**DOI:** 10.1002/1873-3468.70450

**Published:** 2026-09-08

**Authors:** Jack P. K. Bravo

**Affiliations:** ^1^ Institute of Science and Technology Austria

**Keywords:** allostery, Argonaute, guide RNA, guide–target pairing, nucleic‐acid defence, nucleic‐acid surveillance, prokaryotic Argonaute, RNA interference, target recognition, target search

## Abstract

Argonaute proteins mediate RNA interference in eukaryotes and nucleic‐acid defence in prokaryotes. Although these systems operate in distinct biological contexts and produce diverse downstream outcomes, they share a conserved structural architecture and mechanism of guide‐dependent target recognition. Here, we argue that Argonautes function as programmable surveillance platforms that kinetically sample nucleic acids, engage targets through sequential pairing and conformational checkpoints, and couple recognition to functional outputs through regulated structural transitions. Rather than representing distinct recognition mechanisms, the diverse targeting behaviours observed across Argonaute families reflect different implementations of this shared framework, in which guide biogenesis, target accessibility, pairing requirements and effector coupling are weighted differently according to biological context. Viewing Argonautes through this common operational logic reconciles apparent differences in specificity, mismatch tolerance and target selection across systems, while providing a unified conceptual framework for understanding their evolution and guiding the engineering of programmable nucleic‐acid recognition technologies.

## Graphical abstract

Argonaute proteins use short nucleic‐acid guides to locate and regulate specific targets across all domains of life. Despite striking diversity—from human gene silencing to bacterial immune defence—all Argonautes share a conserved three‐stage recognition logic: guide‐directed sampling, progressive target pairing with a conformational checkpoint and system‐specific effector activation. One ancient scaffold, many biological outcomes.

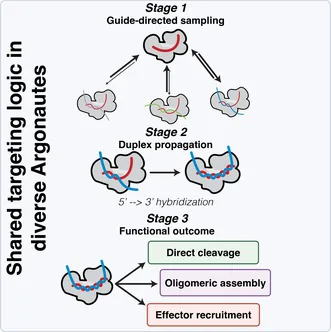

## Abbreviations

Ago, Argonaute

AGO, Argonaute subfamily of eukaryotic Argonautes

AGO2, Argonaute 2

AddAB, ATP‐dependent helicase–nuclease complex involved in double‐strand break repair

CbAgo, *Clostridium butyricum* Argonaute

CRISPR, Clustered Regularly Interspaced Short Palindromic Repeats

DNA, Deoxyribonucleic acid

DdmDE, DdmD–DdmE defence system

DdmD, Helicase–nuclease effector protein of the DdmDE system

DdmE, Catalytically inactive short Argonaute of the DdmDE system

DREN, DNase effector nuclease domain (associated with SPARDA)

dsDNA, Double‐stranded DNA

eAGO/eAgos, Eukaryotic Argonaute(s)

FWF, Austrian Science Fund

GTSF1, Gametocyte‐specific factor 1

HCV, Hepatitis C virus

HNH, Histidine–Asparagine–Histidine nuclease domain

INS, Insertion domain of DdmE

ISTA, Institute of Science and Technology Austria

miRNA, MicroRNA

mRNA, Messenger RNA

NAD(P), Nicotinamide adenine dinucleotide (phosphate)

nt, Nucleotide(s)

pAgo/pAgos, Prokaryotic Argonaute(s)

PAM, Protospacer adjacent motif

PfAgo, *Pyrococcus furiosus* Argonaute

PIWI, P‐element‐induced wimpy testis (Argonaute subfamily)

piRNA, PIWI‐interacting RNA

RecBCD, ATP‐dependent helicase–nuclease complex involved in homologous recombination and double‐strand break repair

RNA, Ribonucleic acid

siRNA, Small interfering RNA

SPARTA, Short prokaryotic Argonaute/TIR‐APAZ defence system (proper system name)

SPARDA, Short prokaryotic Argonaute–DREN defence system (proper system name)

SPARHA, Short prokaryotic Argonaute–HNH defence system (proper system name)

SPARSA, Short prokaryotic Argonaute defence system (proper system name)

ssDNA, Single‐stranded DNA

TDMD, Target‐directed microRNA degradation

TIR, Toll/interleukin‐1 receptor domain

TIR‐APAZ, Toll/interleukin‐1 receptor–APAZ effector module

TldAgo, *Thermococcus litoralis* Argonaute

TtAgo, *Thermus thermophilus* Argonaute

UTR, Untranslated region

Yb body, a cytoplasmic piRNA biogenesis compartment in *Drosophila*

Argonaute proteins are central to nucleic acid‐guided regulation (Fig. 1A) and defence across all domains of life [1, 2, 3]. In eukaryotes, they mediate RNA interference, shaping gene expression, transposon silencing and developmental programmes through small RNA guides [4]. In prokaryotes, Argonautes have been implicated in defence against mobile genetic elements, as well as more recently recognized roles in genome replication, homologous recombination and chromosome maintenance [5]. These roles are typically viewed as functionally distinct, reflecting the ancient ancestry of Argonautes and the diverse biological contexts in which they operate. Yet, this separation obscures a deeper unity: across domains, Argonautes share a conserved structural core and rely on guide‐dependent recognition of nucleic‐acid targets.

**Fig. 1:**
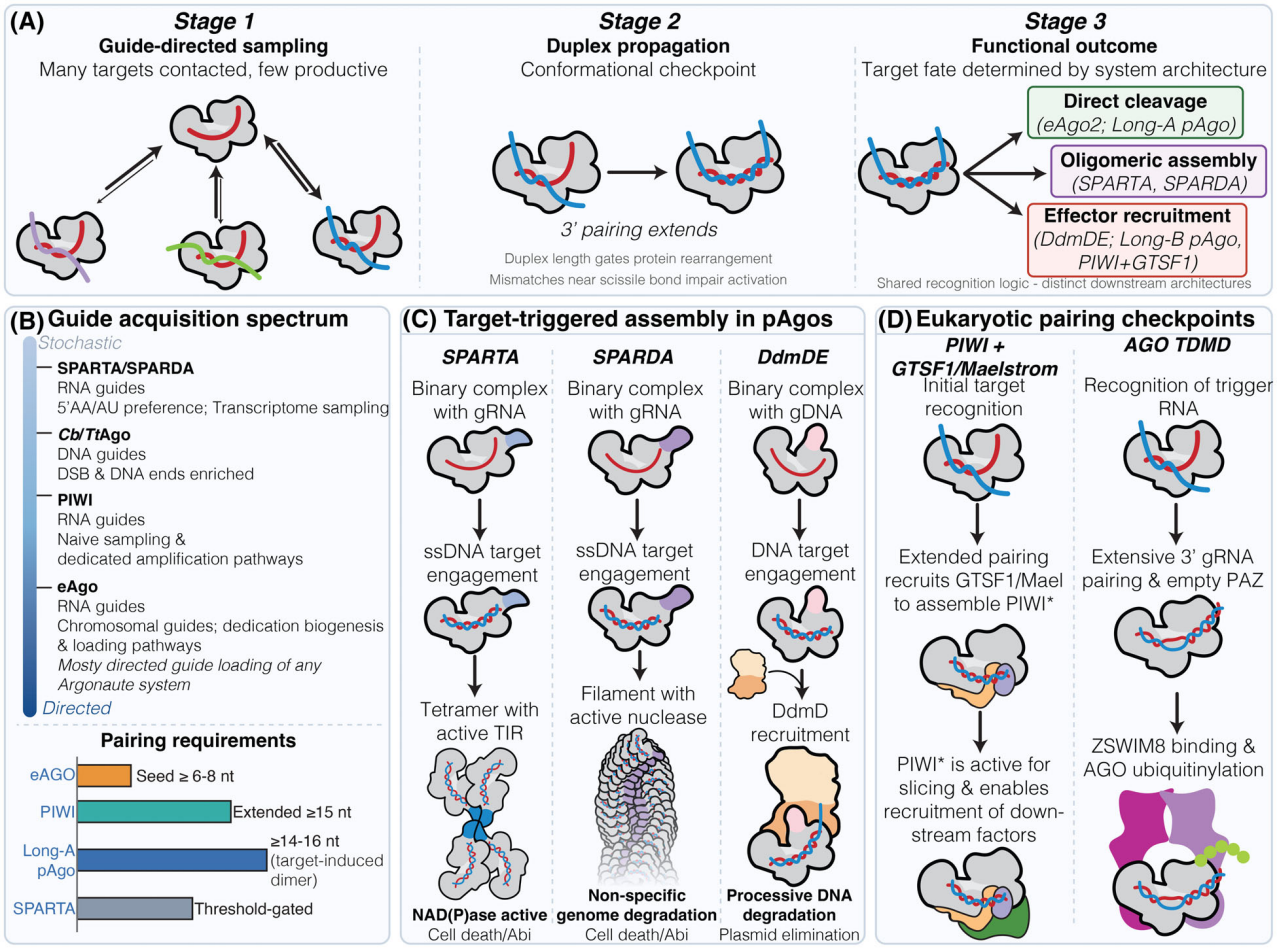
Shared targeting logic underlies Argonaute diversity across domains of life. (A) Argonaute proteins execute a common three‐stage recognition process regardless of system or domain. Stage 1 (guide‐directed sampling): guide‐loaded Argonautes make rapid, reversible seed contacts with potential targets; most interactions are nonproductive and dissociate. Guide composition determines which sequences are encountered. Stage 2 (duplex propagation and conformational checkpoint): productive seed contacts enable 3′ duplex extension, which drives a conformational transition from an open, inactive state to a closed, active state that exposes the effector interface. Mismatches near the scissile bond (Positions 10–15) disproportionately impair this transition even in otherwise mismatch‐tolerant systems. Stage 3 (effector coupling): the activated conformation enables three broad classes of output—direct cleavage (eAGO2, long pAgos), oligomeric effector assembly (SPARTA, SPARDA) or effector recruitment (DdmDE, PIWI+GTSF1)—determined by system architecture rather than the recognition mechanism itself. (B) Guide acquisition spans a spectrum from stochastic to directed. *Top*: SPARTA loads RNA guides predominantly through transcriptome sampling, with a modest 5′ AA preference. SPARDA shows partial 5′‐AU enrichment. CbAgo acquires DNA guides preferentially from RecBCD‐processed double‐strand break sites. TtAgo loads DNA guides enriched at the replication terminus. Eukaryotic AGO and PIWI proteins receive guides from dedicated biogenesis pathways (Dicer cleavage, Yb‐body sorting) with 1 U/10A nucleotide selection and ping‐pong amplification. *Bottom*: pairing requirements across systems. eAGO operates via seed‐dominant binding (≥ 6–8 nt); PIWI‐clade proteins require extended complementarity (≥ 15 nt) with no strict seed; long pAgos require ≥14–16 nt for target‐induced dimerization; SPARTA/SPARDA operate through a density‐dependent threshold. Callout: single G:U wobble in the seed reduces target affinity ≥ 6‐fold in *Drosophila* Ago1, illustrating the extreme seed fidelity of some AGO‐clade proteins. (C) Three prokaryotic systems illustrate how target engagement drives higher‐order assembly. *SPARTA*: target‐bound binary complex oligomerizes into a TIR‐APAZ tetramer, activating NAD(P)ase activity in a density‐dependent threshold switch. *SPARDA*: the apo complex is an autoinhibited dimer with inactive DREN nuclease domains; target engagement drives dimer dissociation, activated monomers assemble into filaments, and DREN domains form tetramers and execute nonspecific dsDNA cleavage, triggering abortive infection. A single target‐recognition event can thus propagate into a population‐level cytotoxic response. *DdmDE*: DdmE (catalytically inactive short pAgo) loads 5′‐phosphorylated DNA guides and engages ssDNA targets; the INS domain recruits autoinhibited dimeric DdmD; and target engagement drives DdmD dimer‐to‐monomer transition, releasing autoinhibition and enabling processive DNA translocation and degradation. All three systems couple single target‐recognition events to amplified or irreversible downstream outputs. (D) Eukaryotic Argonautes employ analogous pairing‐length checkpoints. *PIWI + GTSF1/Maelstrom* (left): cleavage competence requires duplex extension beyond the seed; GTSF1 contacts the target at Positions t21‐23 and Maelstrom contacts t23‐25, collectively acting as a conformational gate that prevents premature cleavage on short or mismatched substrates. Pachytene piRNAs (of which ~99% have no detectable biological target) illustrate that target selectivity in this pathway is determined primarily by downstream filtering rather than guide complementarity alone. *AGO + TDMD* (right): trigger RNAs bearing central mismatches and extensive 3′ complementarity induce an AGO2 conformational change that promotes ubiquitination and miRNA degradation, clearing the guide from the Argonaute. Both pathways demonstrate that duplex geometry, not complementarity alone, determines functional outcome.

Despite this conservation, a persistent challenge has been to reconcile how Argonautes achieve target specificity in complex cellular environments. In eukaryotes, short ‘seed’ matches can drive widespread, often imperfect targeting, while highly complementary sites may be ignored depending on context [6]. In PIWI pathways, for example, extensive complementarity is not required for cleavage, and mismatches can be tolerated throughout the guide–target duplex [7]. As in eukaryotic Argonautes, seed pairing in prokaryotic systems accelerates target search. However, in the prokaryotic Argonautes characterized thus far, progression to stable target engagement and productive activity generally requires more extensive guide–target pairing, with cleavage or effector activation remaining highly dependent on guide–target composition [8]. These observations are often treated as system‐specific peculiarities, but they point to a more general principle: target recognition is not a single binding event dictated by complementarity, but a sequence of partially decoupled steps governed by kinetic, thermodynamic and conformational constraints.

This Innovational Perspective is not intended as a comprehensive review of the rapidly expanding Argonaute literature. Instead, building on several recent authoritative reviews [1, 2, 3, 5, 9, 10, 11], we focus on the common mechanistic principles that emerge across diverse Argonaute systems. We propose a unified framework in which Argonautes function as programmable surveillance platforms that perform biased, guide‐directed sampling of nucleic acids. From this perspective, guide features, Argonaute conformational states and the cellular environment collectively impose ‘prejudices’ that shape which targets are encountered, engaged and acted upon. Crucially, Argonautes do not encode specificity in base pairing alone. Instead, specificity emerges from how individual systems weigh different stages of the targeting pathway—search, binding and execution—to balance precision, robustness and efficiency. This perspective reconciles apparent differences in targeting behaviour and provides a common conceptual basis for understanding Argonaute biology across domains.

## Guides as molecular memory…or products of naïve sampling

A defining feature of Argonaute systems is their reliance on short nucleic‐acid guides, which provide the information required for target recognition (Fig. 1) [2]. The origins of these guides differ substantially across domains (Fig. 1B). In eukaryotes, they are often generated through dedicated biogenesis pathways, such as Dicer processing or transcription from specialized loci, and are selectively loaded into Argonaute proteins [12]. In prokaryotes, guides can arise from degradation products of cellular or foreign nucleic acids, including plasmids and phages, or through broader sampling of the transcriptome [13, 14, 15]. Despite these differences, a unifying principle emerges: [11, 12] guide populations reflect the nucleic‐acid environment of the cell. However, recent work suggests that this relationship is not strictly archival. In PIWI‐interacting RNA (piRNA) systems, guide repertoires can originate from pervasive, largely indiscriminate sampling of cellular transcripts, with specificity emerging only after this initial step through selective processing and amplification [11, 16, 17]. Thus, what appears as molecular ‘memory’ may in part be an emergent property of downstream filtering rather than a predefined record of past exposure.

This mode of guide acquisition contrasts sharply with CRISPR adaptation. In CRISPR systems, dedicated adaptation machinery captures, processes and integrates DNA fragments into genomic CRISPR arrays, creating a heritable record of previous encounters. Although naïve adaptation may incorporate self‐derived DNA, spacer acquisition is shaped by multiple molecular biases that favour foreign DNA and is fundamentally distinct from the passive guide generation observed in Argonaute systems. Instead, recent work on piRNA biogenesis shows that antisense transposon insertions—whether into canonical clusters or into expressed host gene 3′ UTRs—can directly trigger piRNA production through their transcription as part of cellular RNAs [18]. As illustrated by the *tirant* invasion, even a single antisense insertion within a transcribed locus is sufficient to generate piRNAs and establish silencing [19]. In this framework, immunity does not arise from targeted acquisition, but from the intrinsic vulnerability of transposons to be transcribed in antisense orientation within host transcripts. Thus, while piRNA pathways can exhibit adaptive, immune‐like behaviour at the population level, this adaptability emerges from stochastic insertion and transcript sampling rather than from a dedicated acquisition machinery. Conceptually, this process resembles guide generation in many prokaryotic Argonaute systems, where the composition of the guide pool likewise reflects the availability of nucleic‐acid precursors and their preferential processing by cellular nucleases. In both cases, specificity emerges through biased sampling and downstream filtering, rather than through an explicit sequence‐selective acquisition step.

Importantly, guide pools are not neutral representations of sequence space. Their composition is shaped by biases in biogenesis, stability and Argonaute loading preferences. Features such as length, nucleotide composition, terminal identities and chemical modifications influence which guides are incorporated and retained [9]. In some systems, additional layers further skew this landscape: sequence composition biases, RNA processing factors or amplification cycles can selectively enrich particular guide subsets from an initially broad pool. As a result, Argonautes are not simply loaded with random fragments, but with repertoires that are progressively refined, favouring certain targets over others. In this sense, guide populations encode both the availability of sequences and the biases imposed by the molecular machinery that processes them.

In prokaryotic systems, guide acquisition modes span a similar range of bias. Short pAgos, such as SPARTA load RNA guides, predominantly through transcriptome sampling, such that guide abundance broadly reflects transcript abundance. Although several short pAgo systems display modest preferences for particular 5′ nucleotides (for example AA or AU), these loading biases are superimposed on transcriptome‐wide sampling rather than constituting dedicated sequence‐selective guide acquisition mechanisms [20]. The systems show partial compositional preferences: SPARDA and SPARHA preferentially incorporate 5’‐AU RNA guides, indicating that even within the same structural clade the guide‐loading interface can impose partial sequence‐level filtering on top of transcriptome sampling [21, 22, 23].

At the other extreme, DNA‐guided long pAgos exploit the dynamic processes of chromosome replication and DNA repair as sources of acquisition bias [2, 5, 24]. Catalytically active long pAgos, including *Cb*Ago and *Tt*Ago, preferentially acquire guides from chromosome replication termination regions [15] and cooperate with host double‐strand break processing pathways, such as RecBCD or AddAB, reflecting the generation of guide precursors during normal chromosome metabolism [13]. Consistent with these roles, several long pAgos have also been implicated in promoting chromosome replication and counteracting replication stress. Together, these examples illustrate that guide acquisition is shaped by the cellular context in which nucleic‐acid fragments are generated rather than by dedicated sequence‐selective acquisition machinery.

This intrinsic bias has direct consequences for target recognition, but its effects differ across Argonaute systems. In all cases, guide composition skews which sequences are sampled, yet the relationship between guide abundance, target binding and functional outcome is not uniform. In systems such as pachytene piRNAs, only a small minority of guides measurably impact target RNA levels, reflecting limited complementarity, low cleavage efficiency and high target transcription rates [25]. By contrast, eukaryotic AGO proteins can engage large numbers of targets through short seed matches of 6–8 nucleotides, resulting in widespread binding across the transcriptome [4, 26]. However, many of these interactions are transient or weakly repressive, and only a subset produces substantial regulatory effects. Thus, the key distinction is not whether guides have targets, but how many guide–target encounters progress to productive outcomes. Specificity is therefore not determined solely at the level of base pairing, but emerges from a biased, multi‐step filtering process in which guide availability, pairing architecture, catalytic efficiency and target context collectively shape which interactions are ultimately acted upon.

## Target search as a multi‐step, kinetically gated process

Once loaded with a guide, the Argonaute must locate complementary targets within a crowded and noisy nucleic‐acid environment. Rather than relying on exhaustive, high‐affinity binding, Argonautes employ a strategy of rapid sampling combined with sequential filtering [9, 27, 28]. Initial interactions are typically mediated by short stretches of complementarity—most prominently the ‘seed’ region in many systems—which enable fast and reversible contacts with many potential targets. This seed‐driven nucleation accelerates target search but provides only limited discrimination.

Progression beyond this initial encounter requires additional base pairing and is coupled to conformational changes within the Argonaute protein. Guide–target duplex propagation stabilizes the interaction and, in catalytically active systems, promotes rearrangements that position the target strand for cleavage [29].

Recent structural analyses of multiple short prokaryotic Argonaute systems have identified a conserved β‐relay structural element within the PIWI domain that couples guide–target duplex formation to large‐scale conformational rearrangements [21]. Comparative analysis further shows that the same conformational transition is present in the previously determined SPARTA structures, suggesting that β‐relay‐mediated signalling represents a conserved mechanism within this clade of short pAgos. Together, these elements transmit information about guide–target duplex formation to remote protein surfaces, including oligomerization and effector interfaces. These transitions act as checkpoints, allowing Argonautes to discriminate between transient interactions and productive engagements. The stringency of these checkpoints, however, varies across systems: some Argonautes require near‐contiguous pairing for stable binding, whereas others tolerate mismatches throughout the duplex.

This stepwise mechanism implies that productive target recognition cannot be understood from equilibrium binding alone, but instead depends on the dynamic progression through successive binding and conformational checkpoints. Consequently, both the free‐energy landscape of guide–target pairing [6, 8] and the kinetics of target search [27, 28], duplex propagation and conformational activation determine whether a target progresses to a productive outcome.

Weak interactions can be functionally relevant if they occur frequently or persist long enough to trigger downstream effects, whereas perfectly complementary sites may be bypassed if they are rarely encountered or kinetically disfavoured. Moreover, target binding and catalytic activation are not always tightly coupled: in some systems, mismatches can be accommodated—or even enhance cleavage—by promoting conformational states favourable for catalysis. For eAGO2, a combination of central mismatches and extensive 3′ complementarity with a ‘trigger’ RNA activates the target‐directed miRNA degradation pathway culminating in AGO ubiquitinylation and miRNA degradation [30, 31, 32]. Even in systems with broad mismatch tolerance, positional context matters: mismatches flanking the scissile bond (approximately positions 10–15 of the guide–target duplex) disproportionately impair cleavage activation in both PIWI‐clade proteins and related prokaryotic Argonautes, reflecting the requirement for duplex integrity at the catalytic centre to complete conformational gating even when pairing at flanking positions is dispensable [7]. By distributing specificity across multiple stages—encounter, initial pairing, propagation and effector engagement—Argonautes achieve a tunable balance between efficient surveillance and selective action.

## Targets in context: Accessibility and cellular environment

In living cells, potential targets are not freely diffusing linear sequences but are embedded within structured and highly organized nucleic‐acid landscapes. Accessibility is therefore a critical determinant of Argonaute targeting [9, 33]. In prokaryotes, DNA is compacted within the nucleoid and predominantly exists in duplex form, posing a fundamental constraint for DNA‐guided Argonautes that lack intrinsic duplex‐unwinding activity. Biochemical and *in vivo* studies of DNA‐guided pAgos, including *Cb*Ago and *Tt*Ago, indicate that they preferentially engage single‐stranded DNA and are inefficient at invading intact double‐stranded DNA substrates [14, 34].

These observations suggest that although DNA‐guided pAgos can sample genomic sequences broadly, the vast majority of potential targets remain inaccessible because they are embedded within stable duplex DNA. Productive targeting is therefore likely restricted to transient windows of exposure generated during replication, transcription or DNA damage. This raises an important conceptual distinction: guide acquisition may reflect genome‐wide sampling, but functional targeting is constrained by physical accessibility. As a result, the presence of genome‐derived guides does not necessarily imply widespread activity on chromosomal targets, and it remains unclear to what extent self‐targeting guides are able to engage and cleave their cognate loci under physiological conditions.

In eukaryotes, most Argonaute targets are RNA molecules associated with proteins, ribosomes and other regulatory factors. mRNAs adopt secondary and tertiary structures, are translated by ribosomes, and can be sequestered into subcellular compartments, such as processing bodies or stress granules. Each of these features influences which regions are accessible for guide pairing. Notably, partial complementarity within highly stable RNA structures can prevent progression to cleavage despite stable Argonaute binding. In some cases, structured RNA elements can trap Argonaute‐guide complexes in noncanonical conformations, stabilizing binding while occluding the target from nucleolytic attack [33]. Thus, structural context can decouple binding from catalytic outcome, further reinforcing that accessibility and local RNA architecture are critical determinants of productive targeting.

Consequently, the effective target landscape is defined not only by sequence complementarity but also by the spatiotemporal dynamics of nucleic‐acid exposure. Argonautes act opportunistically, engaging targets when they become accessible within the flow of cellular processes. This context dependence helps explain why predicted target sites are not always functional, why binding affinity alone is insufficient to predict targeting outcomes, and why the same guide can produce different effects in different cellular states.

## Same targeting logic, different regimes

The mechanistic diversity of Argonaute systems can be understood not as a collection of distinct targeting rules, but as different operating regimes of a shared molecular framework. Across domains, Argonautes implement the same fundamental sequence of events—target sampling, pairing propagation and effector engagement—but differ in how stringently each step is enforced.

In eukaryotic AGO proteins, targeting operates in an affinity‐dominated regime. Short seed interactions nucleate binding, and the stability of guide–target pairing, modulated by local sequence context and accessibility, determines target occupancy and regulatory output [6]. Specificity emerges from quantitative differences in binding energy distributed across many potential sites, enabling fine‐tuned modulation of gene expression.

By contrast, PIWI proteins function in a tolerance‐dominated regime. Extensive complementarity is not strictly required, and mismatches can be accommodated throughout the guide–target duplex without abolishing cleavage [7]. This relaxed pairing requirement allows PIWI systems to maintain silencing of rapidly evolving transposons, prioritizing robustness over precision.

Prokaryotic Argonautes exhibit an exceptionally broad spectrum of targeting behaviours that reflects their remarkable evolutionary diversity. Rather than occupying a single mechanistic regime, different pAgo systems emphasize distinct combinations of guide acquisition, pairing stringency, conformational activation and effector coupling, illustrating how a common guide‐directed recognition framework can be implemented in multiple ways [35]. Guide acquisition is largely stochastic, while pairing stringency varies from near‐perfect complementarity requirements in long pAgos to more permissive thresholds in short pAgo systems. Effector outcomes are also highly diverse, spanning oligomeric NAD(P)ase activation, filament‐mediated double‐stranded DNA destruction and recruitment of processive helicase–nuclease effectors. Rather than occupying a single ‘intermediate’ position, pAgos illustrate how the same targeting logic can be parameterized across a broad operating range. This spectacular diversity reflects the ancient ancestry of Argonautes and how pAgos have innovated as a consequence of persistent, changing evolutionary pressures over the course of billions of years. Despite their remarkable diversity, prokaryotic Argonautes share a common design principle: guide–target recognition is coupled to downstream conformational transitions that determine whether an encounter progresses to productive activity. In the absence of dedicated guide biogenesis pathways, these postrecognition checkpoints likely play a particularly important role in ensuring that broadly sampled guide populations do not trigger inappropriate effector activation.

Recent structural work reveals that in short pAgo systems, target recognition is coupled to higher‐order assembly. In SPARTA, guide‐dependent target binding drives TIR‐APAZ effector oligomerization via beta‐relay‐mediated conformational change, activating NAD(P)ase activity and imposing a threshold that depends on local target density [20]. The related SPARDA system extends this principle through a structurally elaborated cascade: the apo SPARDA complex is an autoinhibited dimer; guide‐directed ssDNA recognition triggers assembly of activated monomers into filaments to activate a DREN nuclease domain and drive abortive infection [21, 36]. This architecture converts a single target‐recognition event into a population‐level cytotoxic response (Fig. 1C). Related systems, including SPARHA, implement analogous target‐triggered oligomerization cascades through distinct HNH effector nucleases, suggesting that target‐coupled assembly is a recurring design principle within the short pAgo clade [22].

Target‐induced oligomerization is not restricted to catalytically inactive short pAgos. Long‐A pAgos—catalytically active nucleases thought to represent forms ancestral to eukaryotic Argonautes—also undergo target‐induced dimerization. In *Pf*Ago and *Tld*Ago, the binary complex (guide only) is predominantly monomeric; ternary complex formation with ≥ 14 base pairs of guide–target complementarity drives ~90% dimerization and target cleavage [37]. From this perspective, eukaryotic Argonautes—although typically functioning as monomeric regulators—may retain diverged elements of this allosteric architecture in which target recognition continues to be coupled to conformational gating and downstream effector engagement, even if cooperative assembly has been replaced by other regulatory mechanisms.

Together, these observations indicate that Argonaute specificity is not determined solely at the level of individual guide–target interactions, but is further shaped by cooperative assembly mechanisms that integrate multiple recognition events into discrete, thresholded or spatially amplified outputs.

## Conclusions & outlook

Argonautes are best viewed as programmable decision‐making systems that couple nucleic‐acid recognition to downstream outcomes. Across domains of life, they follow a shared logic: guide‐directed sampling of nucleic acids followed by sequential filtering steps that determine whether an interaction is ignored, stabilized or acted upon. What differs between systems is not how targets are found, but how these decisions are executed.

This framework resolves apparent inconsistencies in specificity. Widespread binding, mismatch tolerance and context‐dependent targeting arise naturally from a multi‐step process in which most interactions fail to progress to productive states. Specificity is therefore not dictated solely by complementarity, but emerges from the interplay between guide composition, target accessibility and kinetic thresholds governing progression through recognition checkpoints.

Divergence becomes most apparent at the level of effector coupling. In eukaryotes, Argonautes typically produce local, graded regulatory outputs, modulating transcript stability or translation. In prokaryotes, by contrast, Argonautes are embedded within diverse defence architectures that expand the range of possible outcomes. Some systems convert target recognition into switch‐like, amplified responses, including oligomerization and collateral nucleic‐acid degradation. Others implement direct, processive destruction through modular coupling to auxiliary nucleases. The DdmDE system exemplifies this latter strategy [38, 39, 40]: DdmE, a catalytically inactive short pAgo bearing a unique INS domain, loads 5′‐phosphorylated DNA guides and uses them to engage ssDNA targets; target‐bound DdmE recruits DdmD—an autoinhibited dimeric helicase–nuclease—through a direct handover interaction at the INS domain; target engagement drives a dimer‐to‐monomer transition in DdmD, releasing autoinhibition and enabling processive DNA translocation and degradation (Fig. 1C). The result is targeted, processive elimination of the invading element, coupling single‐guide recognition to complete plasmid destruction.

These observations support a unifying model in which Argonautes act as modular targeting platforms, with effector coupling defining functional output. In this view, recognition and execution are separable layers: the same guide‐directed search process can be wired to distinct outcomes, from fine‐tuned regulation to irreversible DNA destruction.

Underlying this mechanistic diversity is a conserved allosteric architecture. The beta‐relay has now been identified across multiple structurally characterized pAgo clades and transmits guide–target duplex information to remote protein surfaces, including oligomerization and effector interfaces. Its conservation from ancestral long pAgos to the diversified short pAgo clades suggests that target‐coupled conformational activation is an ancient property of the Argonaute fold. Comparable regulatory checkpoints also exist in eukaryotic Argonautes, although they arise through distinct molecular mechanisms (Fig. 1D). For example, PIWI proteins require GTSF1‐ and Maelstrom‐dependent activation to achieve full cleavage competence [41, 42, 43], while AGO proteins undergo target‐induced conformational changes that regulate guide stability through the target‐directed miRNA degradation (TDMD) pathway [30, 31, 32]. Although mechanistically distinct from the β‐relay described in short pAgos, these examples similarly illustrate that guide–target recognition is coupled to downstream regulatory decisions through additional postbinding checkpoints.

Key challenges now lie in defining how these layers are integrated *in vivo*. What determines whether a binding event progresses to activation? How do Argonautes exploit transient windows of target accessibility within structured nucleic‐acid environments? How are guide populations shaped and maintained over time? And how widespread are modular systems that decouple targeting from catalysis?

Emerging studies suggest that some nuclear Argonaute functions may extend beyond canonical small RNA‐guided target recognition, including chromatin‐associated activities whose molecular mechanisms remain incompletely understood [3, 44]. Whether these functions rely on noncanonical guide RNAs, alternative recruitment mechanisms, or both remains an open question. Nevertheless, these observations raise the possibility that the functional repertoire of Argonautes extends beyond classical guide‐directed nucleic‐acid surveillance and represents an important avenue for future investigation.

Answering these questions will be essential for understanding how Argonautes operate as context‐dependent surveillance systems, and how their parameters and outputs can be rewired for biological and technological applications.

## Author contributions

JPKB wrote the perspective.
